# Uterine artery embolization combined with methotrexate for broad ligament ectopic pregnancy in a 30-year old primigravida

**DOI:** 10.1016/j.radcr.2021.05.032

**Published:** 2021-06-15

**Authors:** Khurram Khaliq Bhinder, Azza Sarfraz, Zouina Sarfraz, Shaista Riaz, Samreen Aslam, Zouina Ameena

**Affiliations:** a Shifa International Hospital, Islamabad, Pakistan; b The Aga Khan University, Karachi, Pakistan; c Fatima Jinnah Medical University, Lahore, Pakistan; d McGill University, Montreal, Canada; e Lahore General Hospital, Lahore, Pakistan

**Keywords:** Uterine artery embolization, Ectopic pregnancy, Broad ligament, Obstetrics, Gynecology, Radiology, Pakistan, Case report

## Abstract

We present the case of 30-year old primigravida female at 11 weeks’ gestation who was diagnosed to have ectopic pregnancy on obstetric ultrasound. An MRI pelvis was ordered to assess invasion into the posterior myometrium which confirmed a single right-sided broad ligament-extra uterine pelvic ectopic pregnancy with extrinsic mass effect on the right lower uterine segment without frank myometrial invasion. On an urgent basis, a uterine artery embolization (UAE) was performed by targeting the right femoral artery. Selective catheterization was performed of both uterine arteries and the right side showed a major feeder of the gestational sac. Supplied dose of methotrexate (95mg) was infused in the right uterine artery and both arteries were then embolized by gel foam slurry. Thus, prompt treatment reduced the risk of infertility and saved the patient from obstetrical emergency. Further exploration needs to be done in this field to explore conservative management options to preserve fertility.

## Background

Broad ligament ectopic pregnancy is a rare form of ectopic pregnancy occurring in less than 1% of all ectopic pregnancies [1]. First reported by Loschge in 1816, the incidence of intraligamentary pregnancy is 1 for every 183,900 pregnancies [2]. The foetus or gestational sac develops within the leaves of the broad ligament in the retroperitoneal abdominal cavity. It has frequently been reported in the first trimester yet some cases have not been presented until more advanced gestational ages [3]. Evidence of successful uterine artery embolization (UAE) following systemic methotrexate has been demonstrated in the first trimester for caesarian scar pregnancy [4]. It is difficult to diagnose with no specific clinical features its rare occurrence. The structures surrounding the broad ligament are important to conserve when treating broad ligament ectopic pregnancy. Advanced treatment options of ectopic pregnancies involving minimum invasive procedures include uterine artery embolization (UAE) with systemic methotrexate. UAE has recently emerged as a safe and effective procedure to prevent uncontrollable bleeding and unnecessary uterine loss during obstetric and gynecological emergencies [5].

Here, we present the case of a 30-year old primigravida female with moderate symptoms, diagnosed with a 7.9 × 2.6 × 3.2 cm broad ligament ectopic pregnancy, and managed non-surgically.

## Case presentation

**Fig. 1:**
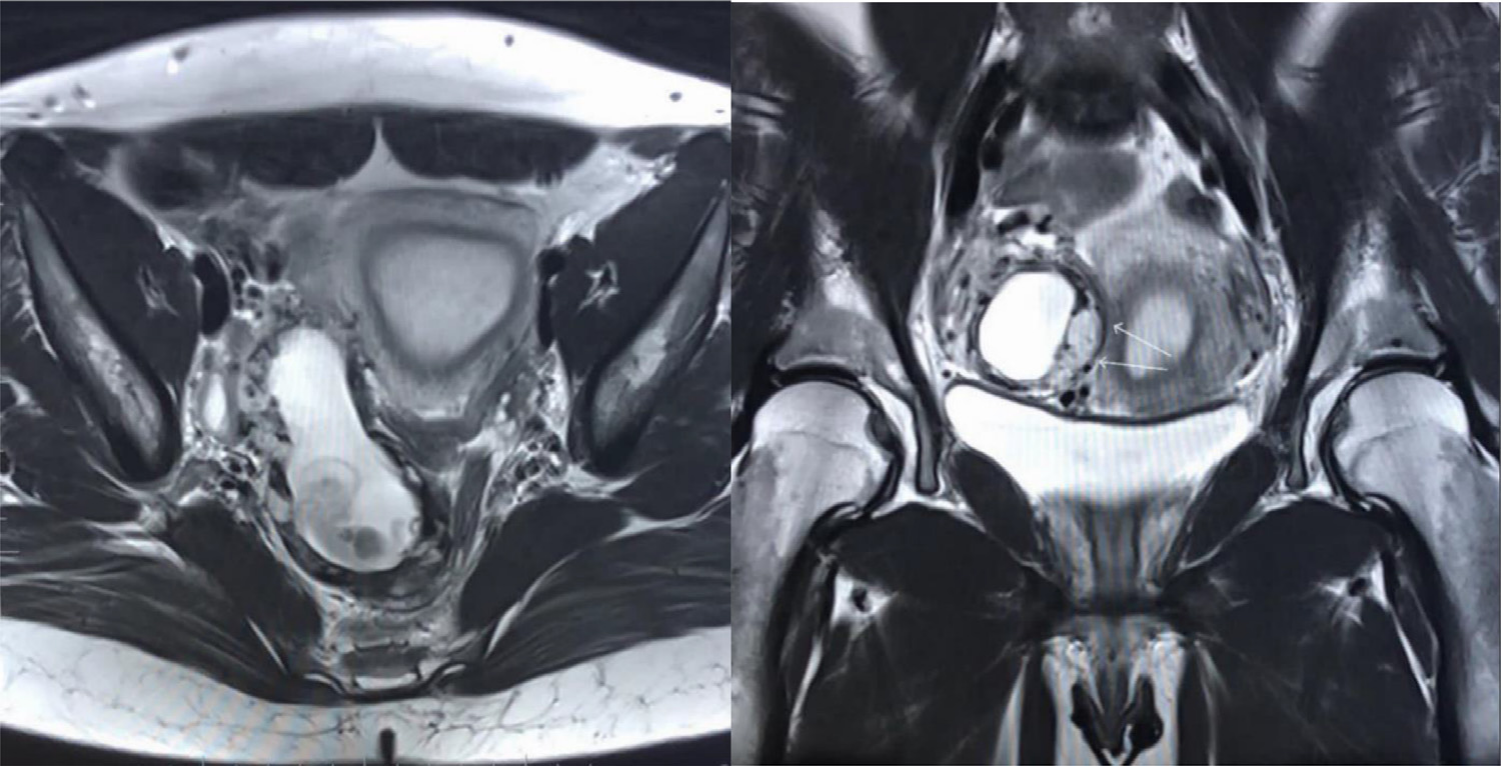
**1a and 1b.** MR axial and coronal view with visible gestational sac.

A 30-year old primigravida female presented to our hospital with pelvic pain associated with weakness and vomiting for 1 week. Considering the patient's history, an urgent obstetrical ultrasound was performed which remarked this to be ectopic pregnancy. Further workup by MRI pelvis was suggested. The MRI was performed which confirmed the presence of an extra-uterine ectopic pregnancy, intra-abdominal in origin along the right broad ligament extending into the right pelvic cul-de-sac. The extra-uterine gestational sac measured 7.9 × 2.6 × 3.2cm, extending along the right pelvic sidewall with abnormal placentation along the right infrolateral myometrial wall with prominent regional collateral vessels (Figs. 1**a and** 1**b**). The gestational sac contained a perceptible embryo with a crown rump length of 3.9cm. There was abnormal parasitization and vascular recruitment from the right ovarian artery and gonadal vein. There was no frank myometrial invasion, with a T2 hypo intense rim of ectopic pregnancy seen throughout and along its medial placental interface. This was extrinsic associated mass effect medially, on the right lower uterine segment, with adjacent prominent regional vessels. Corpus luteal cyst of 2.3cm was seen in the right ovary. No free fluid in the pelvis was seen.

**Fig. 2:**
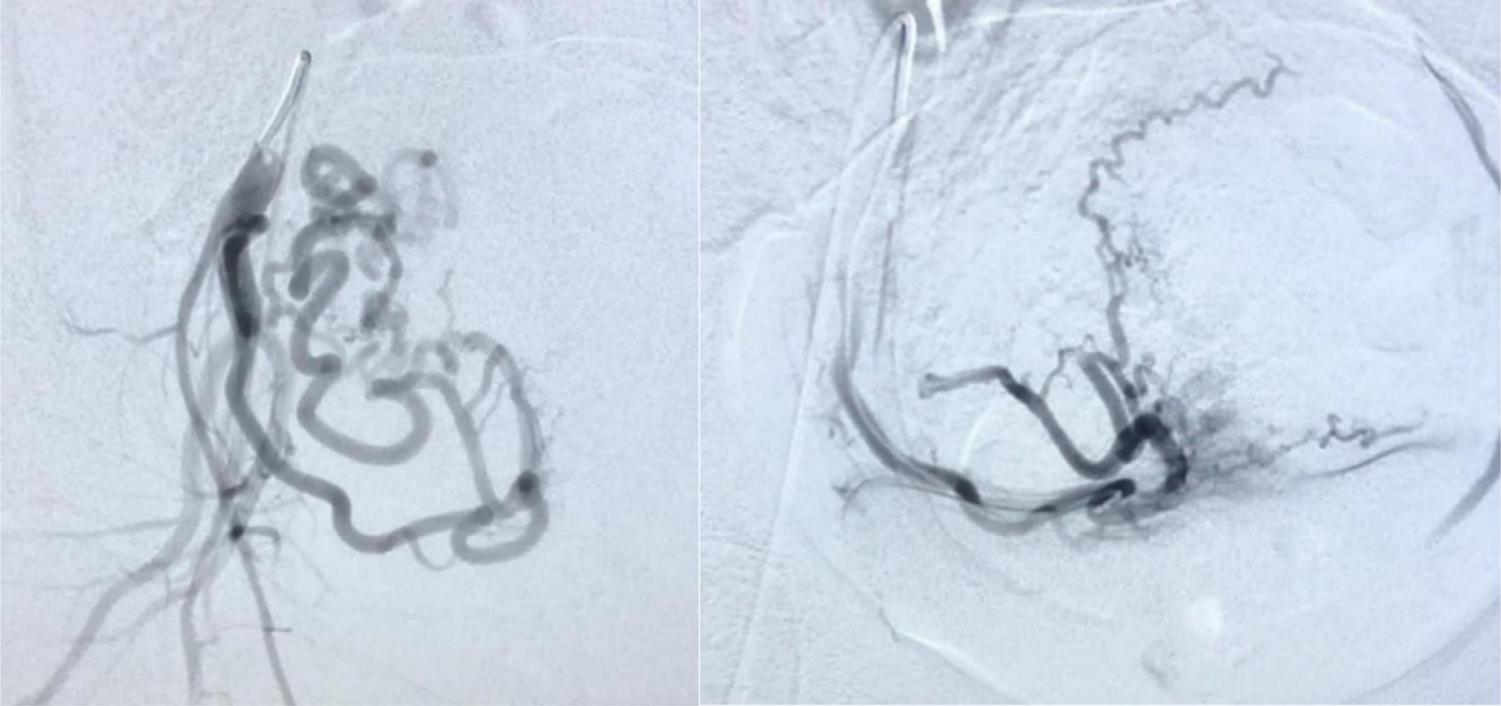
**2a and 2b.** Pre and post embolization of the right uterine artery.

Urgently, uterine artery embolization was performed by targeting the right femoral artery (Figs 2**a and** 2**b**). A 5 Fr regular vascular sheath was placed. A KMP and Progreat microcatheter were used to perform non-selective and selective angiographic images as well as treatment. Selective catheterization was performed on both uterine arteries and the right side showed the major feeder of the gestational sac. Once in stable position, confirmed angiographically, the supplied dose of methotrexate (95mg) was infused in the right uterine artery and both of the arteries were then embolized with gelfoam slurry. The procedure was uneventful with no immediate or late complications noted. The patient was discharged on post procedure day 1 with pain medication, instructions for pelvic rest, adequate hydration, and follow-up in the outpatient clinic with Obstetrics in 2 weeks.

## Discussion

With the intention to preserve the fertility of our patient, a synergistic 2-pronged approach of UAE in combination with systemic methotrexate administration was conducted. We intra-arterially infused methotrexate into the right uterine artery during embolization which showed high therapeutic efficacy. A gelfoam medium was employed for embolization which has shown considerable reduction in the circulation of the catheterized region for up to 24 hours, resulting in recanalization of these vessels 2-6 weeks later^6^. To our knowledge, this is the first case report that reports the efficacy of UAE and intra-arterial methotrexate for a broad ligament ectopic pregnancy with no prior risk factors. There are no set criteria for the mode of therapy among stable patients and approaches are dependent on the patients’ desire to preserve fertility^7^. Conservative management options such as UAE and/or methotrexate during the first trimester in hemodynamically stable patients has advantages including fertility preservation, minimal complications, and reduction of blood loss [6].

The challenge to diagnose early broad ligament pregnancies is due to the overlap of clinical features with other ectopic pregnancies. It may be diagnosed on a prenatal ultrasound as in our case emphasizing the importance of close observation of ultrasonic examination to avoid missing ectopic pregnancies early in the first trimester [8]. When examined closely, ultrasonic evidence can provide the diagnosis, location, and size of the gestational sac; specifically, the presence of an empty uterus separates from the fetus and a placenta situated intra-abdominally provides clear diagnosis [9]. MRI is also a useful adjunct to identify the extent of placental attachments to surrounding vasculature and assess risk of complications [10]. As the broad ligament is anatomically in close proximity to the uterine artery and ureter, the risk of compromising the vasculature is significant, resulting in hemorrhage [11]. So far, studies have reported laparoscopy as the gold standard for early broad ligament ectopic pregnancies [11], [12], [13]. Surgical options are reserved for second, and third trimesters, and complicated first trimester ectopic pregnancies [3,8]. Thus, broad ligament ectopic pregnancies require timely diagnosis and management.

## Conclusion

The management of obstetric emergencies including broad ligament ectopic pregnancy with transcatheter arterial embolization in combination with methotrexate is not common. As a collaborative effort with obstetricians and radiologists, timely diagnosis of abdominal pregnancies in the first trimester with smaller gestational sacs, and hemodynamically stable patients may be managed conservatively with transcatheter intraarterial methotrexate infusion in combination with uterine artery embolization. The patient signed an informed consent form, as per the ethical guidelines of the hospital board.

## Declaration of Competing Interest

The authors declare that the research was conducted in the absence of any commercial or financial relationships that could be construed as a potential conflict of interest.
